# Supplementary material for: Scalable quantum memory in the ultrastrong coupling regime
Source: Sci Rep. 2015 Mar 2;5:8621. doi: 10.1038/srep08621 (PMC4345319; doi:10.1038/srep08621)
Supplement: Supplementary Information — for [file srep08621-s1.pdf]

Supplementary Information for
"Scalable quantum memory in the ultrastrong coupling regime"

T. H. Kyaw,[1] S. Felicetti,[2] G. Romero,[2] E. Solano,[2,3] and L.-C. Kwek[1,4,5]

[1]*Centre for Quantum Technologies, National University of Singapore, 3 Science Drive 2, Singapore 117543, Singapore*

[2]*Department of Physical Chemistry, University of the Basque Country UPV/EHU, Apartado 644, E-48080 Bilbao, Spain*

[3]*IKERBASQUE, Basque Foundation for Science, Maria Diaz de Haro 3, 48013 Bilbao, Spain*

[4]*Institute of Advanced Studies, Nanyang Technological University, 60 Nanyang View, Singapore 639673, Singapore*

[5]*National Institute of Education, Nanyang Technological University, 1 Nanyang Walk, Singapore 637616, Singapore*

(Dated: December 19, 2014)

S1. THE FLUX QUBIT-CAVITY HAMILTONIAN

The potential energy of a compound flux qubit, shown in Fig. S1, is obtained by combining the corresponding Josephson potentials $\mathcal{E}(\varphi_\ell) = -E_{J_\ell}\cos(\varphi_\ell)$, where E_{J_ℓ} and φ_ℓ are the Josephson energy and the superconducting phase across the ℓth Josephson junction (JJ). We assume $E_{J_1} = E_{J_2} = E_J$, $E_{J_3} = \alpha E_J$ and $E_{J_4} = E_{J_5} = \beta E_J$. In addition, the total flux around each closed loop satisfies the flux quantization $\sum_\ell \varphi_\ell = 2\pi f_\ell + 2\pi n$, where φ_ℓ is a gauge-invariant superconducting phase, $f_\ell = \phi_\ell/\phi_0$ is the frustration parameter of the ℓth JJ, and n is an integer-multiple. The potential energy for the free qubit and for the qubit-resonator interaction is given by

$$\begin{aligned}\frac{U}{E_J} =& - [\cos\varphi_1 + \cos\varphi_2 + \alpha\cos(\varphi_2 - \varphi_1 + 2\pi f_1) \\ &+ 2\beta(f_3)\cos(\varphi_2 - \varphi_1 + 2\pi\tilde{f} + \Delta\psi)], \end{aligned} \tag{S1}$$

where $\beta(f_3) = \beta\cos(\pi f_3)$, $\tilde{f} = f_1 + f_2 + f_3/2$ and $\Delta\psi$ stands for the phase slip shared by the coplanar waveguide resonator (CWR) and f_2 loop (see Fig. S1). We also note that the junction at the central line, JJ6, introduces a boundary condition that modifies the mode structure of the cavity but without modifying the potential energy, Eq. (S1). A detailed analysis of an inhomogeneous CWR can be found elsewhere[1,2]. In particular, it has been shown that the phase slip takes the form $\Delta\psi = \Delta\psi_1(a + a^\dagger)$ where $\Delta\psi_1 = (\delta_1/\varphi_0)(\hbar/2\omega_r C_r)^{1/2}$. Here, ω_r is the frequency of the first cavity mode, C_r is the total geometric capacitance of the cavity, $\varphi_0 = \phi_0/2\pi$ is the reduced flux quantum, and $\delta_1 = u_1(x_1) - u(x_2)$ corresponds to a difference between the first-order spatial mode evaluated at the points x_1 and x_2, shared by the resonator and the f_2 loop. The potential U can be further approximated by considering the condition $\Delta\psi_1 \ll 1$, which can be satisfied with realistic

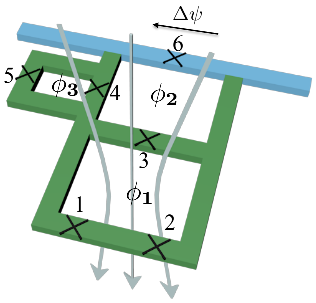

FIG. S1. **The compound flux qubit design**. The JJ6 inserted in the central conductor leads to the ultrastrong coupling and the f_3 loop provides a switchable qubit-resonator coupling strength. Please refer to the text for detailed explanation of the design.

cavity parameters[1,2]. In the following, we make an expansion of $\Delta\psi$ term in Eq. (S1) up to the second order such that we arrive at

$$\frac{U}{E_J} = -\left[\cos\varphi_1 + \cos\varphi_2 + \alpha\cos(\varphi_2 - \varphi_1 + 2\pi f_1) + 2\beta(f_3)\Big[\cos\tilde{\varphi}\Big(1 - \frac{1}{2!}(\Delta\psi)^2\Big) - \Delta\psi\sin\tilde{\varphi}\Big]\right], \tag{S2}$$

where $\tilde{\varphi} = \varphi_2 - \varphi_1 + 2\pi\tilde{f}$. From Eq. (S2), we obtain the flux qubit potential $U_{\text{qubit}} = -E_J(\cos\varphi_1 + \cos\varphi_2 + \alpha\cos(\varphi_2 - \varphi_1 + 2\pi f_1))$ and the tunable qubit-resonator interaction, Eq. (S4). The potential U_{qubit} can be diagonalized numerically as a function of the frustration parameter f_1. In particular, for $f_1 \sim 0.5$, the two lowest energy levels are well separated from higher excited energy levels, thus defining a two-level system. After projecting the qubit-resonator interaction into the qubit basis, the system Hamiltonian reads

$$H_{\text{Rabi}} = \frac{\hbar\omega_{eg}}{2}\sigma_z + \hbar\omega_{\text{cav}}a^\dagger a + H_{\text{int}}, \tag{S3}$$

with the effective interaction Hamiltonian

$$H_{\text{int}} = -\kappa\sum_{n=1,2}(\Delta\psi)^n \sum_{\mu=x,y,z} c_\mu^n(\alpha,\beta,f_1,f_2,f_3)\sigma_\mu, \tag{S4}$$

where $\kappa = 2E_J\beta(f_3)$ and $c_\mu^n(\alpha,\beta,f_1,f_2,f_3)$ are the controllable magnitudes of the longitudinal and transversal coupling strengths for nth order interaction. When external fluxes satisfy $f_2 + f_3/2 = 0.5$, both c_y^1 and the second-order coupling strength are negligible[2]. Thus, the effective interaction Hamiltonian reduces to

$$H_{\text{int}} = \hbar g(a + a^\dagger)(c_z\sigma_z + c_x\sigma_x), \tag{S5}$$

where $c_{z,x} = c_{z,x}^1$ and the switchable qubit-cavity coupling strength $g = 2E_J\beta(f_3)\Delta\psi^1/\hbar$.

S2. CAVITY NETWORK

In order to transfer the state of a qubit along a given path in a cavity network, interactions between neighboring resonators must be selectively turned on and off on demand. This can be done via connecting more resonators to a single circuit node, and then grounding that node through a superconducting quantum interference device (SQUID), as shown in Fig. S2. A SQUID

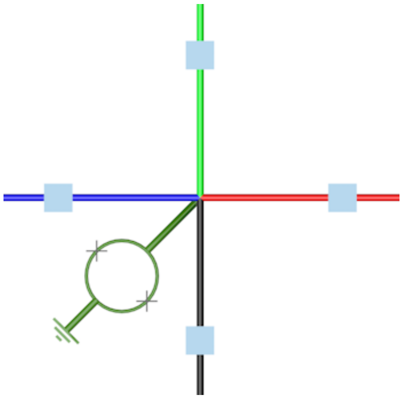

FIG. S2. **A node of the cavity network**, showing four single-mode resonators grounded through a SQUID device. The colour scheme suggests that the frequencies ω_i of the four cavities are different so that direct interactions among them are off-resonance. Hopping interactions between any cavity pair can be activated by driving the SQUID with an external flux, which must be oscillating with the frequency given by sum of the two-cavity bare frequencies.

is a superconducting loop interrupted by two JJs. When a SQUID is perfectly symmetrical, it behaves as a single JJ, such that its effective Josephson energy can be tuned by threading the loop with an external magnetic flux. Indeed, this device can be seen as a tunable inductance, shunted by a small capacitance. The SQUID inductance can be written as $L_J = \frac{\varphi_0^2}{E_J(\phi_{\mathrm{ext}})}$, where $E_J(\phi_{\mathrm{ext}}) = 2E_J \left|\cos\left(\frac{\phi_{\mathrm{ext}}}{2\varphi_0}\right)\right|$. Here φ_0 is the reduced magnetic flux quantum, ϕ_{ext} is the external magnetic flux, and E_J is the Josephson energy. If the system parameters are chosen in order to make the SQUID impedance much smaller than that of the resonators, the electrical potential at the node can be approximated to zero. Such condition is naturally satisfied in most circuit QED experiments involving SQUIDs in the non-dissipative regime. This enables us to define well separated spatial modes for the electromagnetic field in the different resonators. A direct coupling between resonators is then given by the inductive energy term of the SQUID. The strength of this interaction depends on the SQUID impedance and the external flux threading the device. After quantization, the Hamiltonian describing a single network node can be written as (see Ref. 3 for detailed derivation)

$$\begin{aligned}\mathcal{H} = \quad & \hbar \sum_l \omega_l a_l^\dagger a_l \\ & - \hbar \sum_{l,r} \alpha_{l,r}(t) \left(a_l^\dagger + a_l\right) \left(a_r^\dagger + a_r\right), \end{aligned} \tag{S6}$$

where ω_l is frequency of the lth resonator and the indexes l, r run over all resonators pairs. The corresponding coupling parameters are given by

$$\alpha_{l,r}(t) = \frac{\varphi_0^2}{E_J(\phi_{\text{ext}})} \sqrt{\frac{\omega_l \omega_r}{C_l C_r}} \frac{1}{Z_l Z_r}, \tag{S7}$$

where C_i and Z_i indicate the resonator effective capacitance and impedance, respectively. When the resonators are off-resonance, i.e. when $|\omega_l - \omega_r| \gg \alpha_{l,r}$, their interaction is negligible, as far as the coupling strength is constant. Nevertheless, the SQUID can be driven by an external magnetic flux oscillating at frequency comparable with that of the resonators[4]. In this way, the direct interaction term between any resonator pair can be tuned on resonance when the corresponding frequency matching conditions are satisfied. In particular, we can set $\phi_{\text{ext}}/2\varphi_0 = \bar{\phi} + \Delta \cos(\omega_d t)$, where $\bar{\phi}$ is a constant offset and Δ is the amplitude of a small harmonic drive, whose frequency is given by ω_d. Neglecting terms of the order of Δ^2, we obtain

$$\frac{1}{E_J(\phi_{\text{ext}})} \approx \frac{1}{\cos\bar{\phi}} + \frac{\sin\bar{\phi}}{\cos^2\bar{\phi}} \Delta \cos(\omega_d t). \tag{S8}$$

To activate hopping interactions $(a_l^\dagger a_r + a_l a_r^\dagger)$ between two resonators, the driving frequency must satisfy the condition $\omega_d = |\omega_l - \omega_r|$. By controlling the external magnetic flux threading the SQUID device, this scheme allows to selectively turn on an inductive coupling between any two neighboring resonators, which would not interact otherwise.

S3. OPEN QUANTUM SYSTEM DYNAMICS

It is inevitable that any practical and realistic quantum system operates in noisy environment and so is our memory element. Moreover, it is well-known that the standard quantum optics master equation technique is not valid for any value of the qubit-field coupling[5]. Hence, we follow a perturbative expansion of the system-bath coupling strength in the microscopic derivation[6] and obtain the master equation[7]

$$\dot{\rho}(t) = i[\rho(t), H_S] + \mathcal{L}_a \rho(t) + \mathcal{L}_\sigma \rho^\sigma(t), \tag{S9}$$

where $H_S = H_{\text{Rabi}}$ of Eq. S3 and $\sigma = x, y, z$. $\mathcal{L}_a$ and $\mathcal{L}_\sigma$ are Liouvillian superoperators with

$$\begin{aligned} \mathcal{L}_\nu \rho(t) = & \sum_{j,k>j} \Gamma_\nu^{jk} (1 + \bar{n}_\nu(\Delta_{kj}, T)) \mathcal{D}[|j\rangle\langle k|] \rho(t) \\ & + \sum_{j,k>j} \Gamma_\nu^{jk} \bar{n}_c(\Delta_{kj}, T) \mathcal{D}[|k\rangle\langle j|] \rho(t), \end{aligned} \tag{S10}$$

where $\nu = a, x, y, z$, $\mathcal{D}[\mathcal{O}]\rho = (2\mathcal{O}\rho\mathcal{O}^\dagger - \rho\mathcal{O}^\dagger\mathcal{O} - \mathcal{O}^\dagger\mathcal{O}\rho)/2$, T is the temperature of the thermal bath and $\bar{n}_\nu(\Delta_{kj}, T))$ is the number of thermal photons feeding the system from all the possible $|k\rangle \to |j\rangle$ transitions. Here, states $|j\rangle$ are eigenstates of H_{Rabi} with respective eigenenergy $\hbar\omega_j$, i.e., $H_S|j\rangle = \hbar\omega_j|j\rangle$. To arrive at the numerical simulation shown in Fig. 5 of the main text, we assume our system is in a very low temperature environment, i.e., $T \simeq 0$, and the relaxation coefficients take the form[7] $\Gamma_\nu^{jk} = \gamma_\nu \frac{\Delta_{kj}}{\omega_0} |C_{jk}^\nu|^2$, where γ_ν are standard weak coupling damping rates, $\Delta_{kj} = \omega_k - \omega_j$, and $C_{jk}^\nu = -i\langle j|\Theta|k\rangle$, ($\Theta = a - a^\dagger$) for $\nu = a$ and ($\Theta = \sigma_{x,y,z}$) for $\nu = \{x, y, z\}$, respectively.

---

1 Bourassa, J. *et al*. Ultrastrong coupling regime of cavity QED with phase-biased flux qubits. *Phys. Rev. A* **80,** 032109 (2009).

2 Romero, G., Ballester, D., Wang, Y. M., Scarani, V. & Solano, E. Ultrafast Quantum Gates in Circuit QED. *Phys. Rev. Lett.* **108,** 120501 (2012).

3 Felicetti, S. *et al*. Dynamical Casimir effect entangles artificial atoms. *Phys. Rev. Lett.* **113,** 093602 (2014).

4 Wilson, C. M. *et al*. Observation of the dynamical Casimir effect in a superconducting circuit. *Nature* **479,** 376 (2011).

5 Beaudoin, F., Gambetta, J M. & Blais, A. Dissipation and ultrastrong coupling in circuit QED. *Phys. Rev. A*. **84,** 043832 (2011).

6 Breuer, H.-P. & Petruccione, F. *The Theory of Open Quantum Systems* (Oxford Univ. Press, Clarendon, 2006).

7 Ridolfo, A., Leib, M., Savasta, S. & Hartmann, M. J. Photon Blockade in the Ultrastrong Coupling Regime. *Phys. Rev. Lett.* **109,** 193602 (2012).
